# Impact of Dietary Interventions during Pregnancy on Maternal, Neonatal, and Child Outcomes in Low- and Middle-Income Countries

**DOI:** 10.3390/nu12020531

**Published:** 2020-02-19

**Authors:** Zohra S Lassi, Zahra A Padhani, Amna Rabbani, Fahad Rind, Rehana A. Salam, Jai K Das, Zulfiqar A Bhutta

**Affiliations:** 1 Robinson Research Institute, University of Adelaide, Adelaide 5005, Australia; 2 Department of Pediatrics, Aga Khan University, Karachi 74800, Pakistan; zahra.feroz@aku.edu (Z.A.P.); amnarabbani@gmail.com (A.R.); dr.fahadrind@gmail.com (F.R.); rehana.salam@aku.edu (R.A.S.); jai.das@aku.edu (J.K.D.); zulfiqar.bhutta@sickkids.ca (Z.A.B.); 3 Centre for Global Child Health, SickKids Hospital, Toronto, ON M5G 0A4, Canada

**Keywords:** pregnancy, maternal nutrition, balanced energy protein, food distribution program, obesity prevention, maternal, neonatal and child outcomes

## Abstract

Optimal nutrition plays a crucial role in pregnancy. Maternal malnutrition is a risk factor for maternal, fetal, and neonatal complications and is more prevalent in low and middle-income countries (LMICs). This review aims to study the effectiveness of antenatal macronutrient nutritional interventions on maternal, neonatal, and child outcomes. We searched the CENTRAL, PubMed, Embase, and other databases for randomized controlled trials and quasi-experimental designs on healthy pregnant women in LMICs. We also searched grey literature and reports from Google Scholar, Web of Science, and websites of different organizations. Title/abstract screening, full-text screening, and data extraction filtered 15 studies for inclusion. Balanced energy protein (BEP) supplementation (*n* = 8) studies showed a reduced incidence of perinatal mortality, stillbirths, low birth weight (LBW) infants, small for gestational age (SGA) babies and increased birth weight. Food distribution programs (FDPs) (*n* =5) witnessed reduced rates of SGA, stunting, wasting, and increased birth weight and birth length. Studies on intervention for obesity prevention (*n* = 2) showed reductions in birth weight. Other findings were statistically insignificant. Subgroup analyses were conducted to study the effectiveness of supplementation between regions, location, the timing of supplementation and nutritional status; however, there were a limited number of studies in each subgroup. Data from our review supports the antenatal supplementation of BEP and FDP for the prevention of adverse maternal, neonatal, and child outcomes that can be utilized for future policymaking. However, more research is required before recommending obesity prevention programs.

## 1. Introduction

Micro- and macronutrient deficiencies are common among pregnant women in developing countries. These deficiencies contribute to maternal morbidity and mortality [1]. Poor nutrition before and during pregnancy has been associated with maternal, neonatal, and fetal complications, such as intrauterine growth restriction (IUGR), obstructed labor, preeclampsia, anemia, maternal mortality, preterm birth, stillbirth, low birth weight (LBW), neonatal hypothermia, and neonatal mortality [2,3,4,5]. The rates of maternal malnutrition (i.e., body mass index <18.5 kg/m^2^) are greater in low and middle-income countries (LMICs). Several studies suggest the prevalence of maternal malnutrition in LMICs, with one study quoting rates from 20% in sub-Saharan Africa and south/central America to approximately 40% in India [3].

Life course theory demonstrates the childhood experiences affecting health conditions at adulthood (e.g., diabetes, depression) [6]. Likewise, antenatal malnutrition forces a fetus to adapt to an environment of scarcity and, consequently, the adverse effects extend beyond the perinatal period and end up with the child having long-term chronic diseases such as cognitive dysfunction, obesity, diabetes mellitus, and hypertension. Conversely, evidence also suggests the harmful effects of overnutrition [7] in all phases of pregnancy. Nutrition transition has engulfed developing countries, which has caused reduced mortality leading to increased populations, followed by a decrease in fertility. The increase in intake of sugar and fats has also reduced physical activity, contributing to obesity in pregnant women that lead to complicated pregnancies, which also affects the neonate at birth and in future life [8]. It was found that obesity increases the risk of fetal macrosomia, stillbirth, congenital obesity [9,10,11], and infant mortality [12].

Current guidelines have reported on the use and effectiveness of iron and other multi-nutrient formulations, but none of the trials have studied the efficacy of macronutrient supplementation during pregnancy. Although a few trials done over the years have reported on the effect of macronutrient supplementation [13], they are largely in disagreement with each other and no sizable meta-analysis has been performed on these supplementations. A Cochrane review shows the effect of lipid-nutrient supplements on neonatal outcomes in four studies. However, such an expansive investigation into the effect of different types of nutritional supplementation and obesity measures on maternal, neonatal, and child outcomes has not yet been undertaken.

Furthermore, these reviews are not sufficient to derive recommendations for future policies. Thus, due to gap in existing evidence, our review aims to investigate the effect of nutritional interventions during pregnancy on maternal, neonatal, and childhood outcomes.

## 2. Materials and Methods

This systematic review was conducted following the protocol registered on Campbell Systematic Reviews [14].

### 2.1. Objectives

To study the effectiveness of antenatal dietary interventions (balanced energy protein supplementation, food distribution programs, and dietary interventions to prevent maternal obesity) on maternal, neonatal, and child outcomes. We assessed, analyzed, and reported each intervention separately.

### 2.2. Eligibility Criteria

#### 2.2.1. Types of Studies

Our review included individually and cluster randomized controlled trials (RCTs and cRCTs), and quasi-experimental studies, which included natural experiments, controlled before–after (CBA) studies, regression discontinuity designs, and interrupted time series (ITS) series.

#### 2.2.2. Types of Participants

In our review, we included healthy pregnant women living in LMICs, in compliance with the 2018 World Bank definitions [15]. We also included studies in which women were enrolled in the preconception period on a condition that those women were followed up throughout pregnancy. In addition to healthy women, we included both undernourished and obese women without any co-morbids.

#### 2.2.3. Types of Intervention

Our review divided trials along the following definitions of interventions that may or may not benefit pregnant women:

1. BEP supplementation: Food supplementation in which proteins provided less than 25% of the total caloric content [16]
2. Food distribution program (FDP): Internationally or locally-sponsored food distribution programs that provide food to pregnant women and their families. Dietary counseling and education may or may not have been a part of the intervention
3. Interventions for obesity prevention: Interventions aimed at preventing maternal obesity with the utilization of dietary interventions (dietary counseling may or may not be a part of it) only

The comparison groups included standard of care (routine diet).

#### 2.2.4. Types of Outcome Measures

The outcomes in our review were divided into primary and secondary outcomes and then further into maternal, fetal, neonatal, and child outcomes, as expanded below:

1. Primary outcomes
  - Maternal outcomes included body mass index (BMI).
  - Fetal and neonatal outcomes included miscarriage (spontaneous expulsion of a human fetus before it is viable and especially between the 12th and 28th weeks of gestation), stillbirth (baby born with no signs of life at or after 28 weeks’ gestation), perinatal mortality (stillbirth and deaths ≤7 days), and neonatal mortality (death <28 days)
  - Child outcomes included infant mortality (deaths between 0 and 12 months of age) and under-five mortality (deaths between 0 and 59 months).
2. Secondary outcomes
  - Maternal outcomes included maternal mortality (death of a woman while pregnant or within 42 days of termination of pregnancy, irrespective of cause), preeclampsia (as defined by trial authors), placental abruption, overweight (BMI >25 and <30), obesity (BMI >30), and anemia (serum hemoglobin <10.9 g/dL).
  - Fetal outcomes included congenital anomalies.
  - Newborn outcomes included low birth weight (<2500 g), preterm birth (birth at <37 weeks of gestation), small-for-gestational age (World Health Organization), and macrosomia (birth weight >4000 g)
  - Child outcomes included stunting (<-2 Z-score height for age), wasting (<-2 Z-score for weight for height), underweight (<-2 Z-score for weight for age), developmental outcomes (different scales for psychomotor development, attention, memory, language), respiratory disease, allergic disease, hemoglobin concentration (in g/dL), and iron deficiency anemia.

#### 2.2.5. Duration of Follow-Up

Studies with eligible participants and outcomes of interest were included in the review. No limitations were imposed based on the duration of the follow-up, exposure and time of outcome assessment. Outcomes reported at different time points were included following the definition of outcomes. The authors were contacted where the time of follow-up was not given. Data of the longest follow up was captured for childhood and adulthood outcomes.

#### 2.2.6. Type of Settings

Our review only included trials conducted in LMICs. The World Bank defines lower-income countries as countries having a gross national income (GNI) per capita of USD 1005 or less and middle-income countries as countries with a gross national income per capita between USD 1006 and 3955 in 2016 [15].

### 2.3. Literature Search

For this review, we searched the following electronic databases in April 2019:

Cochrane Controlled Trials Register (CENTRAL), MEDLINE, EMBASE, CINAHL, PsycINFO, ERIC, Sociofiles, HMIS (Health Management Information Consortium), CAB Global Health (https://www.cabi.org/publishing-products/online-information-resources/global-health/), the WHO nutrition databases (http://www.who.int/nutrition/databases/en/), Popline (https://www.popline.org), Epistemonikos (https://www.epistemonikos.org/en/), Social Science Citation Index, Dissertation Abstracts International, and WHO Global Health Index, which covers the WHO Regional journals from Latin America (LILACS), Africa (AFRO), among others. We also searched the web sites of selected development agencies or research firms (for example, JOLIS, IDEAS, IFPRI, NBER, USAID, World Bank, and Eldis.org). The trials registry, Clinicaltrials.gov, and WHO’s ICRTP were searched for ongoing trials (Table S1).

Unpublished or ongoing studies were identified by contacting the relevant organizations. Google Scholar and Web of Sciences were searched for citations. We scanned references of included articles, relevant reviews, and bibliographies for eligible studies. We also searched for grey literature on

- Nutrition International (NI)
- Global Alliance for Improved Nutrition (GAIN)
- World Food Programme (WFP)
- UNICEF
- Emergency Nutrition Network (ENN)
- International Food Policy and Research Institute (IFPRI)
- WHOLIS (WHO library database)
- WHO Reproductive Health Library

### 2.4. Data Extraction and Analysis

In our review, data extraction and analysis were conducted according to the Cochrane Handbook for Systematic Reviews of Interventions [17].

Our review focused on experimental study designs (randomized controlled trials and quasi-experimental trials that included controlled before-and-after (CBA) studies).

Before attempting data extraction, we assured compatibility for comparison. Data extraction was divided by the intervention groups specified in the protocol [14]. If studies referred to old data as a part of their analysis, it was cross-checked to prevent repeated analysis of the datasets. Similarly, multi-arm studies had their arms combined for analysis or divided to prevent repeated counting of individual participants. Outcomes that were reported at different metrics between studies were equated to the order of the unit conversions.

Primarily, three investigators (ZAP, AR, and FR) conducted the data extraction and a fourth reviewer was consulted on queries and disagreements. The following information was extracted from the studies:

- Background: Time of study, publication type, study location(s), funding source(s), and conflicts of interest.
- Population and setting: Population age and setting of the study.
- Methods: Study design, description of study arms, unit of allocation, sample or cluster size per study arm, start and end date, and follow-up details.
- Participants: Details of allocation and demo-graphic data.
- Intervention group: Details of allocation, description of the intervention, duration and frequency of the intervention and follow-up, and training level of providers.
- Comparison group: Details of allocation to groups, description of control, duration and frequency of control, follow-up, and training of providers.
- Outcomes: Unit of measurement, values of interest, and results of analyses.
- Other information: Funding, study limitations, and conflict of interests.

A data extraction sheet was designed on an Excel sheet, which was pilot tested before its usage. The sheet allowed for the recording of characteristics of the studies such as author, title, type of study, date of publication, journal of publication, study location, participants’ demographics, study population, details of the intervention, outcomes, inclusion/exclusion criteria, and risk of bias. The dichotomous data were extracted recording the number of events and the number of participants. For continuous data, the mean values and standard deviations (SDs) were recorded. If they were not available, the (SDs) were derived using standard methods. Only intervention arms that conformed to our eligibility criteria of an intervention were included and analyzed in our review.

#### 2.4.1. Assessment of Risk of Bias in Included Studies

The risk of bias (ROB) was assessed by three review authors (ZAP, AR, and FR) and disagreements were resolved by consultation with the fourth reviewer. For RCTs, the Cochrane Collaboration Risk of Bias tool [17] was utilized. Studies were judged to have high, low, or an unclear risk of bias in the following domains, with justifications presented from within the text of the study:

- Random sequence generation
- Allocation concealment
- Blinding of participants and personnel
- Blinding of outcome assessment for each outcome
- Incomplete outcome data
- Selective outcome reporting
- Other bias such as the validity of outcome measure and baseline comparability

For non-randomized controlled trials, EPOC methods [18] were utilized to judge ROB as either low, high or unclear risk of bias in the following domains, with justifications provided from within the text of the study:

- Random sequence generation
- Allocation concealment
- Baseline outcome measurements similar
- Baseline characteristics similar
- Incomplete outcome data
- Knowledge of the allocated intervention adequately prevented during the study
- Protection against contamination
- Selective outcome reporting
- Other risks of bias

#### 2.4.2. Measures of Treatment Effect

The outcome data extracted from the articles were analyzed in RevMan 5.3 [19] and yielded the treatment effects. The effect on dichotomous outcomes was represented by the risk ratio (RR). The effect of continuous outcomes reported in uniform units was represented as mean difference (MD) and those reported in non-uniform adjusted units were represented as standardized mean difference (SMD). A 95% confidence interval was utilized as uncertainty in all effect estimates. If means and SDs were not available, their derivation was attempted using methods outlined in the Cochrane Handbook for Systematic Reviews of Interventions [17]. In data extraction, repeat counting of participants was avoided by assignment to sub-groups in the same plots. Skewing of data due to a small sample size was anticipated and appropriately handled.

#### 2.4.3. Unit of Analysis Issues

Each outcome was meta-analyzed individually. All effect sizes were reported in the study and no outcomes were prioritized over another. Cluster randomized trials’ data was only accepted once adjusted for clustering effects (for example, variance, inflated standard errors, and hierarchical linear models). If there was uncertainty about the cluster adjustment of data, the study authors were contacted for clarification on the nature of the data or the intraclass correlation coefficient (ICC). Using the ICC values, effect sizes and standard errors were meta-analyzed in RevMan 5.3 [19] using the generic inverse method [17] and were then compared with individually randomized trials.

#### 2.4.4. Dealing with Missing Data

If missing data was encountered, the study authors were contacted for further classification; where such contact did not yield clarity, we listed the abstract as a “study awaiting classification”. In case of missing numerical data, study authors were contacted for further classification; where such contact did not yield clarity, we used other values, such as *p*-values, to derive data using the methods described in the Cochrane Handbook for Systematic Reviews of Interventions [17].

#### 2.4.5. Assessment of Heterogeneity

Heterogeneity between studies was assessed using Tau^2^, I^2^ and chi^2^ values, as calculated from RevMan 5.3 [19]. Heterogeneity was further investigated through subgroup analyses.

#### 2.4.6. Assessment of Reporting Biases

Funnel plots were drawn to investigate relationships between effect size and precision, if the study number was sufficient, i.e., more than 10 studies. However, our intervention groups did not cross 10 studies so no funnel plots were drawn. Publication bias was judged by comparing the results extracted from published journal reports with results from other sources (including correspondence).

#### 2.4.7. Data Synthesis

Tables outlining the differences between studies of the same intervention groups were drawn. Meta-analyses were random-effects due to, e.g., the diverse study settings, participants, interventions. Each comparison was compared along with characteristics such as setting, the timing of intervention, duration of intervention, people delivering the intervention.

A “Summary of Findings” table was created using Grading of Recommendations Assessment, Development and Evaluation (GRADE) criteria [20]. GRADE covers the assessment of intra-study sources of bias (methodological quality), the directness of evidence, heterogeneity, the precision of effect estimates, and risk of publication bias. We rated the certainty of evidence for each key outcome as “high”, “moderate”, “low”, or “very low” (Table S2). Randomization started at a default “high” level of evidence whereas lack of randomization defaulted a study to “low” quality of evidence. If studies presented large magnitudes of effect, dose response relationships and effect of plausible residual confounding, or were free of methodological sources of bias, their status was upgraded.

Our review utilized GRADE to construct the summary of findings tables of the following outcomes:

- Stillbirth defined as baby born with no signs of life at or after 28 weeks’ gestation
- Perinatal mortality (stillbirth and deaths ≤ 7 days)
- Neonatal mortality (death < 28 days)
- Infant mortality (deaths between 0 and 12 months)
- Under-five mortality (deaths between 0 and 59 months)
- Low birth weight (<2500 g)
- Preterm birth (<37 weeks gestation)
- Mean maternal body mass index

#### 2.4.8. Assessment of Quality of Evidence

Our review conducted subgroup analyses on primary outcomes where the number of studies allowed such analysis. Subgroup differences were judged based on chi^2^ values as derived on RevMan 5.3. *p*-values of <0.1 were considered significant for heterogeneity. Reasons for heterogeneity were investigated to assess the generalizability of the effects of the intervention. Analyses were formulated for differences based on nutritional status, geographical location, setting, and duration of supplementation, specifically as listed below.

1. Nutritional status: undernourished (BMI < 18.5 vs. well-nourished (BMI > 18.5) pregnant women based on BMI (for BEP and FDP)
2. Region: Africa vs. South Asia vs. South America and the Caribbean
3. Duration of supplementation: whole pregnancy vs. second trimester vs. third trimester
4. Nutritional Status: normal weight vs. overweight vs. obese (for interventions on maternal obesity)
5. Location: rural vs. urban vs. mixed

#### 2.4.9. Subgroup Analysis and Investigation of Heterogeneity

Our review included sub-group analyses conducted on RevMan 5.3. All subgroup analyses used chi^2^ to test heterogeneity and *p*-values of < 0.1 were considered significant. The subgroups analyses aimed to compare the impact of interventions on pregnant women based on their nutritional status, geographical location, region, and duration of supplementation with the following specifications:

1. Nutritional Status: undernourished (BMI < 18.5) vs. well nourished (BMI > 18.5) pregnant women defined based on BMI
2. Region: Africa vs. South Asia vs. South America and the Caribbean
3. Duration of supplementation: whole pregnancy vs. second trimester vs. third trimester
4. Nutritional Status: normal weight vs. overweight vs. obese (for interventions on maternal obesity)
5. Location: rural vs. urban vs. mixed

#### 2.4.10. Sensitivity Analysis

Sensitivity analyses were performed on the primary outcomes of the review to assess the impact of the following standards:

1. Allocation concealment (adequate versus inadequate and/or unclear)
2. Attrition bias (< 10% versus ≥ 10%)
3. Imputed inter correlation coefficients that have been derived in different ways

## 3. Results

### 3.1. Study Selection

We identified 15,983 records after searching different databases. After the removal of 96 duplicates, all records were imported on Covidence for screening. On abstract screening, 61 articles were selected for full-text screening and, at the same time, eight articles were included through cross-referencing. Fifteen studies [21,22,23,24,25,26,27,28,29,30,31,32,33,34,35] were included for data extraction (Figure S1). All included trials studied healthy pregnant women at varying gestational ages in LMIC settings. Eight studies were included in the balanced energy protein (BEP) supplementation category, five studies were placed into food distribution programs (whole food supplementation with dietary education), and two in interventions for obesity prevention trials. 

Studies included both urban [26,28,32,35] and rural [21,23,24,25,29,31,33,34] settings, with three studies not specifying their setting [22,27,30]. The characteristics of included studies are briefly mentioned in Table S3.

### 3.2. Balanced Energy Protein (BEP) Supplementation

#### 3.2.1. Description of Studies

Eight studies on BEP supplementation were included (food supplementation in which proteins provided <25% energy) [21,22,23,25,28,30,32,33], which included pregnant women (Table S3). Three studies were conducted in Asia [21,30,32], three in Africa [22,23,33], one in Middle East [28], and one in South America [25]. Five were randomized controlled trials (RCTs) [21,22,28,32,33], and three were quasi-experimental studies [23,25,30]. 

The studies on BEP assessed macronutrient supplementation in various forms. Some studies provided BEP in form of biscuits [33], while other studies provided it in form of energy. All studies provided supplementation during pregnancy; however, the timing and duration of supplementation varied from the first trimester until the last trimester of pregnancy.

#### 3.2.2. Risk of Bias

Sequence generation was adequately done in one study [33], making the risk of bias low. Stratified design [33] was used for randomization. The randomization method was not clearly mentioned in two trials [22,32], due to which the risk of bias was unclear. The risk of bias was high in two studies due to sequence randomization, increasing the risk of predictability [21,28] (Figure S2). Allocation concealment was not adequately done in one trial, making the risk of bias high [28]. The method of allocation was not properly described in detail in four studies [21,22,32,33]. All six studies failed to report on blinding of participants, personnel, and outcome assessors making the risk of bias unclear. Three studies were at low risk for attrition bias [21,32,33]. One study did not adequately report incomplete outcome data [28], and one study was at high risk of bias due to >10% loss to follow-up [22]. Five studies [21,22,28,32,33] were at unclear risk of selective reporting bias. All five trials were free from other sources of bias.

Three quasi-experimental studies [23,25,30] were judged for a risk of bias using EPOC. The risk of bias for sequence generation was high in all three studies [23,25,30]. The risk for allocation concealment was unclear in one study [23], and high in two studies [25,30]. Baseline outcome measurement and baseline characteristics were similar across the group in one study [23], and was unclear in two studies [25,30]. ROB for incomplete outcome data was low in one study [23], and unclear in two studies [25,30]. Adequate protection against contamination was low in one study [23] and high in two studies [25,30]. The risk of bias for selective outcome reporting was unclear in three studies [23,25,30]; however, ROB was low for other risk of bias.

#### 3.2.3. Effect of BEP Supplementation

BEP supplementation may significantly effect on peripartum deaths with reduced incidences of stillbirths (RR 0.39, 95% CI 0.19 to 0.80, random effect, three studies, 1913 participants; heterogeneity: chi^2^ *p* 0.80, I^2^ 0%, low quality on GRADE) [25,32,33] (Tables S4 and S5; Figure S3).

When this outcome was subdivided on the basis of nutritional status, an insignificant reduction was observed in the rate of stillbirths in studies conducted on well-nourished women (RR 0.45, 95% CI 0.20 to 1.04, random effect, one study, 1446 participants) [33] and on undernourished women (RR 0.27, 95% CI 0.07 to 1.05, random effect, two studies, 467 participants) [25,32]. There was no difference between the well-nourished and undernourished women on stillbirths (RR 0.39, 95% CI 0.19 to 0.80, random effect, three studies, 1913 participants; heterogeneity: chi^2^ *p* 0.80, I^2^ 0%). The subgroup analysis on the basis of geographical setting, location, and duration of supplementation in different trimesters of pregnancy shows similar results as the subgroup analysis on the basis of nutritional status.

In addition, BEP supplementation may show 40% reduction in the incidence of low birth weight (LBW) births (birth weight < 2500 g) (RR 0.60; 95% CI 0.41 to 0.86, random effect, three studies, 1830 participants; heterogeneity: chi^2^ *p* 0.25, I^2^ 27%, low quality of evidence on GRADE) [23,32,33]. Anthropometric effects of BEP were reported to have a significant increases in birth weight (MD 107.28, 95% CI 68.51 to 146.04, random effect, eight studies, 2190 participants; heterogeneity: chi^2^ *p* 0.13, I^2^ 35%) [21,22,23,25,28,30,32,33], and an insignificant increase in birth length (MD 0.28; 95% CI −0.36 to 0.92, random effect, two studies, 67 participants; heterogeneity: chi^2^ *p* 0.77, I^2^ 0%) [21,32]. Similarly, a 29% significant decrease in the incidence of small-for-gestational-age births (RR 0.71; 95% CI 0.54 to 0.94, random effect, five studies, 1844 participants; heterogeneity: chi^2^ *p* value: 0.28, I^2^ 21%) [23,25,30,32,33]. Dwarkanath 2016 [32] and Mora 1978 [25] reported no impact of BEP supplementation on preterm births (RR 0.86, 95% CI 0.50 to 1.46, random effect, two studies, 467 participants; heterogeneity: Chi² P value: 0.54, I² 0%, very low quality on GRADE). 

Other outcomes were not reported by more than one study, so they could not undergo meta-analysis. Dwarkanath [32] was the only study to report the incidence of miscarriage but the results did not find any difference between the intervention and control groups (RR 1.00, 95% CI 0.07 to 14.21, random effect, one study, 24 participants). One study reported on perinatal, neonatal, and infant mortality [33]. The study reported that BEP supplementation may reduce incidence of perinatal mortality (RR 0.50, 95% CI 0.30 to 0.84; random effect, one study, 1446 participants; very low quality on GRADE). BEP supplementation may not have had any effect on neonatal mortality (RR 0.58; 95% CI 0.32 to 1.06; random effect, one study, 1446 participants; moderate quality of evidence on GRADE), and infant mortality (RR 1.00, 95% CI 0.59 to 1.68; one study, random effect, 1446 participants; moderate quality of evidence on GRADE). Ceesay (1997) [33] also reported on the impact of BEP supplementation on head circumference, with the effect being significant (MD 3.1 mm, one study, *p*-value < 0.01, SE: 0.11).

Studies on BEP did not report on a few primary outcomes such as under-five mortality and on secondary outcomes such as maternal mortality, pre-eclampsia, placental abruption, overweight, obesity, maternal anemia, iron deficiency anemia in mothers, congenital anomalies, macrosomia, stunting, wasting, and underweight.

We did not exclude any of the trials on BEP supplementation when performing sensitivity analysis to consider the impact of attrition bias, since all of the studies reporting primary outcomes had low attrition bias, thus having no effect on any primary outcome. However, when conducting sensitivity analysis for allocation concealment we had Mora 1978 [25] with high risk of allocation concealment, reporting primary outcomes. When conducting sensitivity analysis by excluding Mora 1978 [25], no significant change was observed in stillbirths.

For the sensitivity analysis of imputed inter-correlation coefficients (ICC), we did have one trial [33] for which cluster adjustment was done by applying ICC, derived from Leroy (2016) [27]. So, we conducted a sensitivity analysis by excluding Ceesay (1997) [33] and studying its impact on all the primary outcomes. There was a significant change in results of stillbirth (from RR 0.39, 95% CI 0.19 to 0.80, random effect, three studies, 1913 participants; heterogeneity: chi^2^ *p* 0.80, I^2^ 0%; to RR 0.27, 95% CI 0.07 to 1.05, random effect, two studies, 467 participants; heterogeneity: chi^2^ *p* 0.87, I^2^ 0%).

### 3.3. Food Distribution Programs

#### 3.3.1. Description of Studies

We included five trials, providing food to pregnant women (Table S3) [24,27,29,31,34]. Out of five studies, three were conducted in Africa [27,29,34], and two in Asia [24,31]. Three of these were cRCTs [24,27,34], and two were RCTs [29,31]. The food programs provided nutritional supplement along with food rations [27], free medical care, such as antenatal/postnatal care [31], maternity services or obstetrical care [24], malaria treatment [34], and HIV counseling [34], to all pregnant women.

#### 3.3.2. Risk of Bias

Sequence generation was adequately done by five trials [24,27,29,31,34]. Methods of randomization included stratified design [24], block randomization [29,34], factorial design, and a lottery event [27]. Out of five studies, only two studies had adequately discussed the method of allocation concealment [29,34]. One study used opaque envelopes [34] while one study mentioned the use of numbered boxes [29]. Three trials did not clearly mention the method of allocation concealment [24,27,31]. Blinding of participants was adequately done in one study, indicating a low risk of bias [34]. Blinding of participants was inadequately done in two studies, leading to a high risk of performance bias in Mridha (2016) and Johnson (2017) [24,29]. Two studies had unclear risks of bias because these studies [27,31] failed to report on blinding. Blinding of outcome assessors was adequately done by three studies [24,29,34], and was not clearly mentioned in two studies [27,31]. One study had <10% attrition, making the risk of bias low [34]. One study [27] failed to report on loss to follow-up, resulting in an unclear risk of bias. Rate of attrition was >10% in three studies, leading to a high risk of bias [24,29,31]. One study showed a low risk of reporting bias [31], which were consistent between protocol, methodology and practices, whereas, one study had an unclear risk of reporting bias [29]. Three trials did not report all outcomes, leading to a high risk of selective reporting bias [24,27,34]. No other sources of bias were made apparent after data extraction.

#### 3.3.3. Effect of Food Distribution Programs

Food distribution programs may be associated with a 33% decrease in perinatal mortality (RR 0.67; 95% CI 0.41 to 1.09, random effect, two studies, 4852 participants; heterogeneity: chi^2^ *p* 0.29, I^2^ 11%, low quality on GRADE; Figure S4, Tables S4 and S6; [24,34]).

When using subgroups for geographical settings, an insignificant reduction was reported in the rate of perinatal mortality in studies conducted in Africa (RR 0.45, 95% CI 0.19 to 1.09, random effect, one study, 841 participants) [34], and Asia (RR 0.78, 95% CI 0.47 to 1.32, one study, random effect, 4011 participants) [24]. There was no difference between Africa, and Asia for perinatal mortality (RR 0.67; 95% CI 0.41 to 1.09, random effect, two studies, 4852 participants; heterogeneity: chi^2^ *p* 0.29, I^2^ 11%).

An insignificant reduction in maternal mortality was reported by two studies (RR 0.41, 95% CI 0.07 to 2.49, random effect, two studies, 4925 participants; heterogeneity: chi^2^ *p* value 0.79, I^2^ 0%) [24,34]. Food distribution program may have no effect on preterm births (RR 0.92; 95% CI 0.78 to 1.10, random effect, three studies, 4608 participants; heterogeneity: chi^2^ *p* value 0.56, I^2^ 0%, low quality on GRADE) and SGA (RR 0.94; 95% CI 0.89 to 1.00, random effect, three studies, 4511 participants; heterogeneity: chi^2^ *p* 0.84, I^2^ 0%) [24,29,34].

The combined results of four studies also revealed that food distribution program probably show an 8% decrease in the rates of low birth weight infants (RR 0.92, 95% CI 0.84 to 1.00, random effect, four studies, 5552 participants; heterogeneity: chi^2^ *p* 0.85, I^2^ 0%, moderate quality on GRADE) [24,29,31,34]. Birth weight was a frequently reported outcome with in three studies reporting figures on it. Food programs reported a significant 46 g increase in birth weight (MD 46.00, 95% CI 45.10 to 46.90, random effect, three studies, 5272 participants; heterogeneity: chi^2^ *p* 0.83, I^2^ 0%) [24,31,34]. 

Birth length was also frequently reported and resulted in a mean 0.2 cm increase (MD 0.20, 95% CI 0.20 to 0.20, random effect, three studies, 5272 participants; heterogeneity: chi^2^ *p* value 0.46, I^2^ 0%) [24,31,34]. The impact of food distribution programs on the anthropometric measurement of head circumference was reported by two studies (MD 0.07, 95% CI −0.22 to 0.36, random effect, two studies, 4490 participants; heterogeneity: chi^2^ *p* 0.003, I^2^ 88%) [24,31].

Food program trials also reported a statistically significant 18% reduction in the incidence of stunting (RR 0.82, 95% CI 0.71 to 0.94, random effect, two studies, 4166 participants; heterogeneity: chi^2^ *p* 0.72, I^2^ 0%) [24,34]. A 13% reduction in risk of wasting was reported by two studies [24,34], with the overall effect being significant (RR 0.87, 95% CI 0.78 to 0.97, random effect, two studies, 3883 participants; heterogeneity: chi^2^ *p* 0.95, I^2^ 0%). Two trials [24,34] studied the impact of food distribution program on underweight children. However, the overall effect was not significant (RR 0.84, 95% CI 0.63 to 1.13, random effect, two studies, 4174 participants; heterogeneity: chi^2^ *p* 0.19, I^2^ 41%).

Other outcomes were not meta-analyzed as they were only reported by singular studies. Miscarriage was only reported by Mridha (2016) [24] among the FDP trials and did not indicate any significant association (RR 0.89, 95% CI 0.67 to 1.19, random effect, one study, 4011 participants). Infant and neonatal mortality was also reported by one study [34], which shows that the food distribution program may have had no effect on neonatal mortality (RR 0.46; 95% CI 0.20 to 1.04, random effect, one study, 841 participants, low quality on GRADE), and on infant mortality (RR 0.34, 95% CI 0.01 to 8.41, random effect, one study, 841 participants; moderate quality on GRADE). Anemia of pregnant women was only reported by Leroy (2016) [27], which indicated separate associations for the T24 (children who received supplementation until 23.9 months of age; RR 0.70; 95% CI 0.60 to 0.82) and the T18 group (children who received supplementation until 18 months of age; RR 0.79, 95% CI 0.70 to 0.89). Both of the intervention groups revealed significant reductions in the incidences of anemia in pregnant women. 

Studies of FDP did not report on few primary outcomes such as maternal BMI and under-five mortality, and on secondary outcomes such as pre-eclampsia, placental abruption, overweight, obesity, maternal iron deficiency anemia, congenital anomalies, macrosomia, hemoglobin concentration, and iron deficiency anaemia.

We did not exclude any of the trials on FDP when performing sensitivity analyses to consider the impact of allocation concealment on primary outcomes since all studies reporting primary outcomes were unclear about allocation concealment. 

We excluded Mridha (2016) [24] while performing sensitivity analysis to consider the impact of attrition bias, and Imputed Inter Correlation Coefficients (ICC) on perinatal mortality. We did not observe a significant shift in results in perinatal mortality after removing Mridha 2016 [24]. 

### 3.4. Interventions for Obesity Prevention Programs

#### 3.4.1. Description of Studies

Research on obesity prevention programs is comparatively lacking, with only two eligible studies extracted for data totaling 192 participants [26,35] (Table S2). Out of the two included studies, one study was conducted in Asia [26] and one in Europe (Turkey) [35]. One study was a RCTs [35] and one was a quasi-experimental trial [26]. Interventions for obesity prevention was mainly focused on dietary interventions, along with counseling sessions, and behavioral change advice.

#### 3.4.2. Risk of Bias

Sequence generation was adequately done in Asci (2016) [35], making the risk of bias low. In this study randomization, lots were drawn for sequence generation [35]. The method of allocation concealment was not clearly mentioned [35], due to which the risk of bias was unclear. Blinding of participants and personnel was adequately done in this study, indicating a low risk of bias; however, it failed to report on blinding for outcome assessors. This study was at a high risk of attrition bias due to >10% loss to follow-up, but it was at a low risk of bias for selective reporting. The study was also free from other sources of bias [35].

One quasi-experimental trial [26] was judged for the risk of bias using EPOC. ROB for allocation sequence generation, adequate protection against contamination, selective outcome reporting was low, along with other risks of bias. Baseline outcome measurement and baseline characteristics were similar across the group. The risk of bias for allocation concealment, adequate protection against contamination, and prevention of knowledge of allocated intervention was unclear; however, ROB was high for incomplete outcome data.

#### 3.4.3. Effect of Obesity Prevention Programs

The analysis revealed that obesity prevention programs led to a statistically significant reduction in birth weight (MD −195.57, 95% CI −349.46 to −41.68, two studies, 180 participants, heterogeneity: chi^2^ *p* 0.84, I^2^ 0%) [26,35]. Other data resulting from the review were only reported by single studies. One trial reported on the effect of obesity prevention interventions on birth length. However, the results were not significant (MD −0.36, 95% CI −1.12 to 0.40, random effect, one study, 90 participants) [35]. Another study reported a 43% statistically insignificant reduction in the incidence of macrosomia (RR 0.57, 95% CI 0.18 to 1.82, one study, 90 participants) (Table S4) [26]. All the reported outcomes were adjusted for age and education so it did not have an effect on our results.

Studies on intervention for obesity prevention did not report any data on maternal, fetal, newborn, and child primary outcomes and on fetal and child secondary outcomes. These studies also did not report a few maternal and newborn secondary outcomes such as maternal mortality, pre-eclampsia, placental abruption, obesity, iron deficiency anemia, low birth weight, small-for-gestational age, and head circumference of the newborn.

We could not perform a sensitivity analysis of trials on obesity prevention interventions since none of the included studies reported primary outcomes of our interest.

## 4. Discussion

Maternal undernutrition and over-nutrition is a global health issue and a leading cause of maternal, neonatal, and childhood morbidity and mortality worldwide [36]. This systematic review consolidates information on dietary interventions including BEP supplementation, food distribution programs, and obesity prevention interventions targeting healthy pregnant females, filling a gap in knowledge.

In agreement with existing data, our systematic review proves that BEP supplementation is a well-developed method, contributing to the improvement of maternal, neonatal, and child outcomes when evaluated in terms of nutrition status, morbidity, mortality, and biochemical status. This review includes trials conducted in nine low- and middle-income countries spanning four continents, i.e., Asia, Africa, Europe, and South America. The BEP trials were comparatively uniform in the specifications of their interventions (protein less than 25% of the total energy) [16]. Most of the outcomes showed improvement after BEP supplementation (stillbirth, perinatal mortality, birth weight, low birth weight, and SGA), and FDP (low birth weight, birth weight, birth length, stunting, wasting, and SGA) thus ripe for application on policymaking and recommendations. Studies on interventions for obesity prevention showed a reduction in rates of macrosomia, but they cannot be used for evidence generation due to less number of included studies.

Trials on BEP supplementation reported that intervention led to a decrease in rates of low birth weight infants (40%), stillbirths before 30 weeks of gestation (61%), perinatal mortality (50%), SGA babies (29%), and increased mean birth weight. There was no effect of BEP supplementation on mean birth length, preterm birth, infant mortality, and neonatal mortality. Sensitivity analysis was conducted to consider the impact of allocation concealment, attrition bias, and ICC on primary outcomes. The sensitivity analysis for allocation concealment and ICC led to removal of one study from stillbirth outcomes. Sensitivity analysis of allocation concealment did not show any shift in results, however, sensitivity analysis for ICC resulted in a significant change in the outcome of stillbirths. There was no effect of attrition bias on sensitivity analysis.

Food distribution programs showed an improvement in mean birth weight (46 g), birth length (0.20 cm), SGA (18%), stunting (18%), and wasting (13%). There was no impact of food distribution programs on perinatal, neonatal, infant, and maternal mortality, preterm births, mean head circumference, and rates of underweight babies. For sensitivity analysis of FDP, three studies were unclear, and one study was low for selection bias; therefore, none of them were excluded. However, we found no significant change in the results of perinatal mortality when conducting sensitivity analysis for attrition bias, and ICC. Interventions to prevent maternal obesity led to a decrease in mean birth weight (195.57 g) and there was no effect on mean birth length and macrosomia. Also, we were unable to conduct a sensitivity analysis for this intervention group.

Quality of the evidence for the individual studies was judged by using the ROB assessment tool for RCTs and cRCTs, and EPOC for quasi-experimental trials. GRADEpro was used to judge the quality of evidence of the reported primary outcomes. The overall quality of the studies included in our review assessing the effects of BEP supplementation ranged from moderate to very low and food distribution program outcomes ranked from moderate to low. Interventions for obesity prevention studies did not report any of the primary outcomes so we were unable to perform a GRADE analysis. Poor quality of evidence of BEP supplementation, and food program studies based on our GRADE analysis recommends high-quality research to further study the impact of dietary interventions during pregnancy. Since RCTs lack the overall stringency, marking many areas of ROB as unclear and high, thus further trials should report on blindness, follow-up data, method of allocation, and other areas of bias before being accepted.

Similar results were also shown by other systematic reviews. Our study agreed with a recent review [37], which was aimed at assessing the maternal and child benefits of increasing energy and protein supplementation in the diet of pregnant women. Ota (2015) [37] included 11 studies on BEP supplementation, both from LMICs (seven studies) and HICs (four studies). Our results were in concordance with this study as it reported decreased stillbirths and decreased small-for-gestational age. Our discoveries are also in agreement with a systematic review by Imdad (2011) [38] (including six studies from LMICs and five from HICs) to the extent that both studies reported a statistically significant increase in birth weight and a reduction in stillbirths. An another comparable review, Kramer (2003) [39], also concluded sufficient benefits of BEP supplementation to pregnant mothers. We could not find any systematic reviews conducted to study the effect of food distribution programs during pregnancy in LMICs.

Systematic reviews investigating the efficacy of obesity prevention in the last decade have been attempted but proved largely suboptimal with Furber (2013) [40] netting no studies. Of note, all included obesity prevention studies in our review were published in 2016 at the earliest. Flynn (2016) [41] aimed to assess the effects of GDM prevention strategies on maternal and child outcomes; however, the outcomes profile was wholly incomparable to our review due to its emphasis on the participants’ progression of diabetes. This places our review as the first meta-analysis of the effects of obesity prevention programs on the outcome of pregnancy.

During subgroup analysis based on location and regions, we found negligible heterogeneity between urban and rural settings, and between Asia, Africa and South America. This can be used as evidence to support the global generalizability of BEP supplementation and FDP. The significant outcomes reported a beneficial effect of prenatal BEP supplementation and FDP, thus implying the implementation of such intervention at a large scale in LMICs. However, there is a need for further research on interventions for obesity prevention among pregnant women through dietary interventions for evidence generation.

Our study is the first one to report on three interventions, i.e., balanced energy protein supplementation, food distribution programs, and interventions for obesity prevention in one systematic review, as previously no other review has studied the three interventions collectively. Another major strength of this systematic review is the gathering of evidence from the latest research. Moreover, in interventions for obesity prevention studies, in contrast to other trials that reported both the impact of exercise and food on obesity prevention, our study focused only on dietary interventions and its impact on maternal, infant, and child outcomes. In addition, no internal sources of bias were identified during the curation of the study.

The limitations of our systematic review are the small number of studies included, the small number of participants in the majority of the studies, and the differences between the studies regarding population, setting, and type of intervention. Moreover, we did not receive any reply from any of the contacted first authors.

BEP supplementation and FDPs had a significant effect on improving maternal and child outcomes, hence it can be used for evidence generation. In contrast, interventions for obesity prevention studies require more research before recommending its use and putting evidence into practice. Further research is recommended to study the impact of dietary interventions on maternal (pre-eclampsia, placental abruption, anemia), fetal (congenital anomalies), and child (developmental outcomes, respiratory disease, allergic disease, anemia, and iron deficiency anemia) outcomes before making judgments and recommending its use.

## 5. Conclusions

Data from our review supports the antenatal supplementation of BEP and FDPs for the prevention of adverse maternal, neonatal, and child outcomes and can be utilized for future policymaking. However, more research is required before recommending obesity prevention programs. There is also a need for further research on nutritional interventions during pregnancy in low- and middle-income countries for evidence generation.

## Supplementary Materials

The following are available online at https://www.mdpi.com/2072-6643/12/2/531/s1, Figure S1: Search Flow Diagram, Figure S2: Risk of Bias Summary, Figure S3: Forest plot of comparison: Balanced Energy Protein vs. Control, outcome: Stillbirth, Figure S4: Forest plot of comparison: Food Program vs. Control, outcome: Perinatal Mortality; Table S1: Search Strategies, Table S2: Quality of evidence, as determined by GRADE criteria, Table S3: Characteristics of Included Studies, Table S4: Summary of Meta-Analysis, Table S5: Summary of Findings (SoF) Table of Balanced Energy Protein (BEP), Table S6: Summary of Findings of Food Distribution Program (FDP).

## References

[1] Villar J., Merialdi M., Gülmezoglu A.M., Abalos E., Carroli G., Kulier R., de Onis M. (2003). Nutritional Interventions during Pregnancy for the Prevention or Treatment of Maternal Morbidity and Preterm Delivery: An Overview of Randomized Controlled Trials. J. Nutr..

[2] Ahmed T., Hossain M., Sanin K.I. (2012). Global burden of maternal and child undernutrition and micronutrient deficiencies. Ann. Nutr. Metab..

[3] Black R.E., Victora C.G., Walker S.P., Bhutta Z.A., Christian P., de Onis M., Ezzati M., Grantham-McGregor S., Katz J., Martorell R. (2013). Maternal and child undernutrition and overweight in low-income and middle-income countries. Lancet (Lond. Engl.).

[4] Christian P., Mullany L.C., Hurley K., Katz J., Black R. (2015). Nutrition and maternal, neonatal, and child health. Semin. Perinatol..

[5] Zerfu T.A., Umeta M., Baye K. (2016). Dietary diversity during pregnancy is associated with reduced risk of maternal anemia, preterm delivery, and low birth weight in a prospective cohort study in rural Ethiopia. Am. J. Clin. Nutr..

[6] Cheng T.L., Solomon B.S. (2014). Translating life course theory to clinical practice to address health disparities. Matern. Child Health J..

[7] Kimani-Murage E.W., Muthuri S.K., Oti S.O., Mutua M.K., van de Vijver S., Kyobutungi C. (2015). Evidence of a double burden of malnutrition in urban poor settings in Nairobi, Kenya. PLoS ONE.

[8] Rozowski J., Parodi C.G., Lammi-Keefe C.J., Couch S.C., Philipson E.H. (2008). Implications of the Nutrition Transition in the Nutritional Status on Pregnant Women. Handbook of Nutrition and Pregnancy.

[9] Stothard K.J., Tennant P.W., Bell R., Rankin J. (2009). Maternal overweight and obesity and the risk of congenital anomalies: a systematic review and meta-analysis. Jama.

[10] Catalano P., Demouzon S. (2015). Maternal obesity and metabolic risk to the offspring: why lifestyle interventions may have not achieved the desired outcomes. Int. J. Obes..

[11] Alfaradhi M., Ozanne S. (2011). Developmental programming in response to maternal overnutrition. Front. Genet..

[12] Meehan S., Beck C.R., Mair-Jenkins J., Leonardi-Bee J., Puleston R. (2014). Maternal obesity and infant mortality: a meta-analysis. Pediatrics.

[13] Lechtig A., Habicht J.-P., Delgado H., Klein R.E., Yarbrough C., Martorell R. (1975). Effect of food supplementation during pregnancy on birthweight. Pediatrics.

[14] Lassi Z.S., Imdad A., Ranjit D., Saint Surin G.S., Salam R.A., Bhutta Z.A. (2019). PROTOCOL: Effects of nutritional interventions during pregnancy on birth, child health, and development outcomes: A systematic review of evidence from low and middle income countries. Campbell Syst. Rev..

[15] Bank W. New country classifications by income level: 2018-2019. https://blogs.worldbank.org/opendata/new-country-classifications-income-level-2018-2019.

[16] Imdad A., Bhutta Z.A. (2012). Maternal nutrition and birth outcomes: Effect of balanced protein-energy supplementation. Paediatr. Perinat. Epidemiol..

[17] Higgins J.P.T., Altman D.G., Gøtzsche P.C., Jüni P., Moher D., Oxman A.D., Savović J., Schulz K.F., Weeks L., Sterne J.A.C. (2011). The Cochrane Collaboration’s tool for assessing risk of bias in randomised trials. BMJ.

[18] Cochrane Effective Practice and Organisation of Care Suggested risk of bias criteria for EPOC reviews. https://epoc.cochrane.org/sites/epoc.cochrane.org/files/public/uploads/Resources-for-authors2017/suggested_risk_of_bias_criteria_for_epoc_reviews.pdf.

[19] (2014). Review Manager (RevMan) [Computer program]. Version 5.3.

[20] (2015). GRADEpro GDT [Computer program].

[21] Tontisirin K., Booranasubkajorn U., Hongsumarn A., Thewtong D. (1986). Formulation and evaluation of supplementary foods for Thai pregnant women. Am. J. Clin. Nutr..

[22] Ross S.M., Nel E., Naeye R.L. (1985). Differing effects of low and high bulk maternal dietary supplements during pregnancy. Early Hum. Dev..

[23] Prentice A.M., Cole T.J., Foord F.A., Lamb W.H., Whitehead R.G. (1987). Increased birthweight after prenatal dietary supplementation of rural African women. Am. J. Clin. Nutr..

[24] Mridha M.K., Matias S.L., Chaparro C.M., Paul R.R., Hussain S., Vosti S.A., Harding K.L., Cummins J.R., Day L.T., Saha S.L. (2016). Lipid-based nutrient supplements for pregnant women reduce newborn stunting in a cluster-randomized controlled effectiveness trial in Bangladesh. Am. J. Clin. Nutr..

[25] Mora J., Navarro L., Clement J., Wagner M., De Paredes B., Herrera M.G. (1978). The effect of nutritional supplementation on calorie and protein intake of pregnant women. Nutr. Rep. Int..

[26] Liu Y.Q., Liu Y., Hua Y., Chen X.L. (2017). Effect of diet and exercise intervention in Chinese pregnant women on gestational weight gain and perinatal outcomes: A quasi-experimental study. Appl. Nurs. Res. Anr.

[27] Leroy J.L., Olney D. (2016). Tubaramure, a Food-Assisted Integrated Health and Nutrition Program in Burundi, Increases Maternal and Child Hemoglobin Concentrations and Reduces Anemia: A Theory-Based Cluster-Randomized Controlled Intervention Trial. J. Nutr..

[28] Kaseb F., Kimiagar M., Ghafarpoor M., Valaii N. (2002). Effect of traditional food supplementation during pregnancy on maternal weight gain and birthweight. Int. J. Vitam. Nutr. Res..

[29] Johnson W., Darboe M.K., Sosseh F., Nshe P., Prentice A.M., Moore S.E. (2017). Association of prenatal lipid-based nutritional supplementation with fetal growth in rural Gambia. Matern. Child Nutr..

[30] Girija A., Geervani P., Rao G.N. (1984). Influence of dietary supplementation during pregnancy on lactation performance. J. Trop. Pediatr..

[31] Frith A.L., Naved R.T., Persson L.A., Frongillo E.A. (2015). Early prenatal food supplementation ameliorates the negative association of maternal stress with birth size in a randomised trial. Matern. Child Nutr..

[32] Dwarkanath P., Hsu J.W., Tang G.J., Anand P., Thomas T., Thomas A., Sheela C.N., Kurpad A.V., Jahoor F. (2016). Energy and Protein Supplementation Does Not Affect Protein and Amino Acid Kinetics or Pregnancy Outcomes in Underweight Indian Women. J. Nutr..

[33] Ceesay S.M., Prentice A.M., Cole T.J., Foord F., Weaver L.T., Poskitt E.M., Whitehead R.G. (1997). Effects on birth weight and perinatal mortality of maternal dietary supplements in rural Gambia: 5 year randomised controlled trial. BMJ (Clin. Res. Ed.).

[34] Ashorn P., Alho L., Ashorn U., Cheung Y.B., Dewey K.G., Harjunmaa U., Lartey A., Nkhoma M., Phiri N., Phuka J. (2015). The impact of lipid-based nutrient supplement provision to pregnant women on newborn size in rural Malawi: a randomized controlled trial. Am. J. Clin. Nutr..

[35] Aşcı Ö., Rathfisch G. (2016). Effect of lifestyle interventions of pregnant women on their dietary habits, lifestyle behaviors, and weight gain: a randomized controlled trial. J. Health Popul. Nutr..

[36] WHO (2013). Global Nutrition Policy Review: What Does it Take to Scale up Nutrition Action?.

[37] Ota E., Tobe-Gai R., Mori R., Farrar D. (2012). Antenatal dietary advice and supplementation to increase energy and protein intake. Cochrane Database Syst. Rev..

[38] Imdad A., Bhutta Z.A. (2011). Effect of balanced protein energy supplementation during pregnancy on birth outcomes. BMC Public Health.

[39] Kramer M.S., Kakuma R. (2003). Energy and protein intake in pregnancy. Cochrane Database Syst. Rev..

[40] Furber C.M., McGowan L., Bower P., Kontopantelis E., Quenby S., Lavender T. (2013). Antenatal interventions for reducing weight in obese women for improving pregnancy outcome. Cochrane Database Syst. Rev..

[41] Flynn A.C., Dalrymple K., Barr S., Poston L., Goff L.M., Rogozinska E., van Poppel M.N., Rayanagoudar G., Yeo S., Barakat Carballo R. (2016). Dietary interventions in overweight and obese pregnant women: a systematic review of the content, delivery, and outcomes of randomized controlled trials. Nutr. Rev..

