# Supplementary material for: Impact of Dietary Interventions during Pregnancy on Maternal, Neonatal, and Child Outcomes in Low- and Middle-Income Countries
Source: Nutrients. 2020 Feb 19;12(2):531. doi: 10.3390/nu12020531 (PMC7071393; doi:10.3390/nu12020531)
Supplement: Supplementary file 1 [file nutrients-12-00531-s001.pdf]

Tables and Figures

Table S1. Search Strategies

Search Strategies
**PubMed**
Population Strategy – Pregnant Women
"Pregnancy"[Mesh] OR "Gravidity"[Mesh] OR "Pregnant Women"[Mesh] OR "Prenatal Care"[Mesh] OR "Perinatal Care"[Mesh] OR "Prenatal Diagnosis"[Mesh] OR "Obstetrics"[Mesh] OR pregnan*[tiab] OR gravid*[tiab] OR gestat*[tiab] OR (pregnant women[tiab] OR pregnant woman[tiab] OR Periconception[tiab]) OR childbear*[tiab] OR maternity[tiab] OR obstetric*[tiab] OR (prepartum[tiab] OR pre partum[tiab]) OR (antepartum[tiab] OR ante partum[tiab]) OR (prenat*[tiab] OR pre nat*[tiab]) OR (antenat*[tiab] OR ante nat*[tiab]) OR (perinat*[tiab] OR peri nat*[tiab])
Dietary Diversity Strategies
("Diet/methods"[Mesh] OR "Diet/trends"[Mesh] OR "Diet/standards"[Mesh]) OR ("Dietary diversity" OR "diet diversity" OR "Diet quality" OR "Dietary Quality" OR "Dietary Quality Index" OR "Dietary Variety" OR "Diet variety" OR "Dietary Diversity Score" OR "Food Variety Score" OR "Nutritional Diversity" OR "Nutrient diversity" OR "Nutritional Functional Diversity" OR "Nutritional adequacy" OR "Nutrient adequacy" OR "Nutrition adequacy") OR (Dietary Pattern* OR Diet Pattern* OR Nutritional pattern* OR Nutrition pattern*)
Balanced Energy Protein Supplementation
("Energy Intake"[Mesh] OR "Dietary Proteins"[Mesh] OR protein[tiab] OR energy[tiab] AND supplement*)
Food Distribution Programs
("Food assistance"[Mesh] OR "Food assistance" OR "Food distribution" OR "Food aid" OR "Nutrition assistance" OR food program*)
Maternal Obesity Interventions
("Obesity/prevention and control"[Mesh] OR "Weight Gain"[Mesh]) AND "Humans"[Mesh]))
**Embase**
((((('pregnant woman'/exp OR 'pregnant woman' OR 'pregnancy'/exp OR 'pregnancy' OR 'gravidity'/exp OR 'gravidity' OR 'prenatal care'/exp OR 'prenatal care' OR 'perinatal care'/exp OR 'perinatal care' OR 'prenatal diagnosis'/exp OR 'prenatal diagnosis' OR 'obstetrics'/exp OR 'obstetrics' OR 'child bearing'/exp OR 'child bearing' OR 'preconception'/exp OR 'preconception' OR 'prepartum' OR 'periconception' OR 'antenatal') AND 'diet method' OR 'diet standard' OR 'dietary diversity'/exp OR 'dietary

diversity' OR 'diet quality'/exp OR 'diet quality' OR 'diet quality index'/exp OR 'diet quality index' OR 'diet variety' OR 'dietary diversity score'/exp OR 'dietary diversity score' OR 'food variety score' OR 'nutritional functional diversity' OR 'nutritional adequacy' OR 'dietary pattern'/exp OR 'dietary pattern' OR 'diet pattern'/exp OR 'diet pattern' OR 'nutrition pattern'/exp OR 'nutrition pattern' OR 'nutritional pattern') AND ('energy intake'/exp OR 'energy intake') OR 'protein intake'/exp OR 'protein intake' OR 'dietary proteins'/exp OR 'dietary proteins' OR 'protein supplement') AND ('food assistance'/exp OR 'food assistance') OR 'food distribution'/exp OR 'food distribution' OR 'food aid' OR 'nutrition assistance' OR 'food program') AND ('obesity prevention and control'/exp OR 'obesity prevention and control') OR 'body weight gain'/exp OR 'body weight gain') AND ('humans'/exp OR 'humans')

Psych Info

((((('pregnant woman'/exp OR 'pregnant woman' OR 'pregnancy'/exp OR 'pregnancy' OR 'gravidity'/exp OR 'gravidity' OR 'prenatal care'/exp OR 'prenatal care' OR 'perinatal care'/exp OR 'perinatal care' OR 'prenatal diagnosis'/exp OR 'prenatal diagnosis' OR 'obstetrics'/exp OR 'obstetrics' OR 'child bearing'/exp OR 'child bearing' OR 'preconception'/exp OR 'preconception' OR 'prepartum' OR 'periconception' OR 'antenatal') AND ('diet method' OR 'diet standard' OR 'dietary diversity'/exp OR 'dietary diversity' OR 'diet quality'/exp OR 'diet quality' OR 'diet quality index'/exp OR 'diet quality index' OR 'diet variety' OR 'dietary diversity score'/exp OR 'dietary diversity score' OR 'food variety score' OR 'nutritional functional diversity' OR 'nutritional adequacy' OR 'dietary pattern'/exp OR 'dietary pattern' OR 'diet pattern'/exp OR 'diet pattern' OR 'nutrition pattern'/exp OR 'nutrition pattern' OR 'nutritional pattern') AND ('energy intake'/exp OR 'energy intake') OR 'protein intake'/exp OR 'protein intake' OR 'dietary proteins'/exp OR 'dietary proteins' OR 'protein supplement') AND ('food assistance'/exp OR 'food assistance') OR 'food distribution'/exp OR 'food distribution' OR 'food aid' OR 'nutrition assistance' OR 'food program') AND ('obesity prevention and control'/exp OR 'obesity prevention and control') OR 'body weight gain'/exp OR 'body weight gain') AND ('humans'/exp OR 'humans')

CENTRAL

ID Search Hits

#1 MeSH descriptor: [Pregnant Women] explode all trees

#2 'Gravidity'

#3 'pregnant women'

#4 'preconception'

#5 'periconception'

#6 'child bearing'

#7 'obstetrics'

#8 'prepartum'

#9 'antepartum'
#10 'prenatal'
#11 'perinatal'
#12 'Balanced Energy Protein Supplementation'
#13 'Energy Intake'
#14 'Dietary Proteins'
#15 'Dietary Diversity Strategies'
#16 'diet methods'
#17 'diet trend'
#18 'diet standards'
#19 'dietary diversity'
#20 'diet quality'
#21 'dietary quality'
#22 'Dietary Quality Index'
#23 'Dietary Variety'
#24 'Dietary Diversity Score'
#25 'Food Variety Score'
#26 'Nutritional Diversity'
#27 'Nutrient Diversity'
#28 'Nutritional Functional Diversity'
#29 'Nutritional adequacy'
#30 'Nutrition adequacy'
#31 'Dietary Pattern'
#32 'diet pattern'
#33 'Nutrition pattern'
#34 'Food Distribution Program'
#35 'food assistance'

#36 'food distribution'

#37 'nutrition assistance'

#38 'food program'

#39 'Maternal Obesity Intervention'

#40 'Obesity prevention and control'

#41 'weight gain'

#42 humans

#43 #1 or #2 or #3 or #4 or #5 or #6 or #7 or #8 or #9 or #10 or #11 and #12 or #13 or #14 and #15 or #16 or #17 or #18 or #19 or #20 or #21 or #22 or #23 or #24 or #25 or #26 or #27 or #28 or #29 or #30 or #31 or #31 or #32 or #33 and #34 or #35 or #36 or #37 or #38 and #39 or #40 or #41 and #42

MEDLINE

1. 'pregnant woman'/exp or 'pregnant woman'.mp. or 'pregnancy'/exp or 'pregnancy'.mp. or 'gravidity'/exp or 'gravidity'.mp. or 'prenatal care'/exp or 'prenatal care'.mp. or 'perinatal care'/exp or 'perinatal care'.mp. or 'prenatal diagnosis'/exp or 'prenatal diagnosis'.mp. or 'obstetrics'/exp or 'obstetrics'.mp. or 'child bearing'/exp or 'child bearing'.mp. or 'preconception'/exp or 'preconception'.mp. or 'prepartum'.mp. or 'periconception'.mp. or 'antenatal'.mp. [mp=title, abstract, original title, name of substance word, subject heading word, floating sub-heading word, keyword heading word, organism supplementary concept word, protocol supplementary concept word, rare disease supplementary concept word, unique identifier, synonyms]

2. ('diet method' or 'diet standard').mp. or 'dietary diversity'/exp or 'dietary diversity'.mp. or 'diet quality'/exp or 'diet quality'.mp. or 'diet quality index'/exp or 'diet quality index'.mp. or 'diet variety'.mp. or 'dietary diversity score'/exp or 'dietary diversity score'.mp. or 'food variety score'.mp. or 'nutritional functional diversity'.mp. or 'nutritional adequacy'.mp. or 'dietary pattern'/exp or 'dietary pattern'.mp. or 'diet pattern'/exp or 'diet pattern'.mp. or 'nutrition pattern'/exp or 'nutrition pattern'.mp. or 'nutritional pattern'.mp. [mp=title, abstract, original title, name of substance word, subject heading word, floating sub-heading word, keyword heading word, organism supplementary concept word, protocol supplementary concept word, rare disease supplementary concept word, unique identifier, synonyms]

3. 'energy intake'/exp or 'energy intake'.mp. or 'protein intake'/exp or 'protein intake'.mp. or 'dietary proteins'/exp or 'dietary proteins'.mp. or 'protein supplement'.mp. [mp=title, abstract, original title, name of substance word, subject heading word, floating sub-heading word, keyword heading word, organism supplementary concept word, protocol supplementary concept word, rare disease supplementary concept word, unique identifier, synonyms]

4. 'food assistance'/exp or 'food assistance'.mp. or 'food distribution'/exp or 'food distribution'.mp. or 'food aid'.mp. or 'nutrition assistance'.mp. or 'food program'.mp. [mp=title, abstract, original title, name of substance word, subject heading word, floating sub-heading word, keyword heading word, organism supplementary concept word, protocol supplementary concept word, rare disease supplementary concept word, unique identifier, synonyms]

5. ((('obesity prevention.mp. and control'/exp) or 'obesity prevention.mp.) and control'.mp.) or 'body weight gain'/exp or 'body weight gain'.mp. [mp=title, abstract, original title, name of substance word, subject heading word, floating sub-heading word, keyword heading word, organism supplementary concept word, protocol supplementary concept word, rare disease supplementary concept word, unique identifier, synonyms]

6. ('human' or 'humans').mp. [mp=title, abstract, original title, name of substance word, subject heading word, floating sub-heading word, keyword heading word, organism supplementary concept word, protocol supplementary concept word, rare disease supplementary concept word, unique identifier, synonyms]

7. 1 and 2 and 3 and 4 and 5 and 6

8. 1 and 2 and 3 and 4 and 5

Table S2. Quality of evidence, as determined by GRADE criteria

Quality	Description
Very low	Any estimate of effect is uncertain
Low	Further research is very likely to have important impact on our confidence in the estimate of effect and is likely to change the estimate.
Moderate	Further research is likely to have an important impact on our confidence in the estimate of effect and may change the estimate.
High	Further research is very unlikely to change our confidence in the estimate of effect.

Table S3. Characteristics of Included Studies

Intervention group	Study	Country	Intervention	Comparison	Outcomes
**Balanced Energy Protein**	Ceesay 1997[1]	Gambia	2 biscuits daily composed of roasted ground-nuts, rice flour, sugar, groundnut oil, providing a maximum intake of 4250kJ energy (1015kcal), 22g protein, 56g fat, 47mg Calcium and 1.8mg Iron. Intervention lasted 20 weeks and was delivered through birth attendants.	Both groups received: 1. Routine antenatal care (interview; abdominal palpation; blood pressure and hemoglobin and urine protein concentrations; treatment or referral as indicated) from midwives in a mobile clinic that visited each village twice monthly. 2. Iron and folate supplements (according to hemoglobin concentration) 3. Tetanus toxoid to not previously protected women 4. prophylactic dose of chloroquine (in hungry season)	Stillbirths, perinatal mortality, neonatal mortality, infant mortality, low birth weight, birth weight, birth length and head circumference.
	Dwarkanath 2016[2]	India	The supplemented group received a daily dietary supplement of 300kcal/d and 15g protein/d, provided as 3 small, round ‘‘granola-type" treats, called ladoos (made of crushed roasted peanuts, puffed rice, skimmed milk, clarified butter, and unrefined sugar) were provided from the first trimester of pregnancy to delivery.	The control group continued to consume their habitual diet	Miscarriage, low birth weight, preterm birth, small-for-gestational age, birth weight and birth length.
	Girija 1984 [3]	India	Supplement :50 g sesame cake, 40 g jaggery, and 10 g oil (Total energy: 417 kcal)	Routine diet	Nutritional intake, weight gain, haemoglobin level,

				birth weight, birth length, head circumference, and arm circumference
Kaseb 2002[4]	Iran	Received traditional food supplementation everyday of 400kcal and 15g protein from the Ghaemiah health care center. Supplementation was composed of rice-milk porridge, lentils, pottage, cheese, yogurt, eggs, and milk with bread, was given five days during the week. Weight and height were recorded on the first meeting. Intervention was delivered from the 4th month of pregnancy to childbirth.	Was handled similarly with exception of food supplements and lasted from the 4th month of pregnancy to childbirth. (Every enrolled women was provided with prenatal care)	Birth weight and gestational weight gain.
Mora 1978 [3,5-8]	Colombia	Supplement: 60 g dried skim milk, 150 g enriched bread, and 20 g vegetable oil (Total energy: 856 kcal)	No intervention	Stillbirths, SGA, birth weight, preterm birth
Prentice 1987[9]	Gambia	The supplement utilized local foods and readily available food consisting of groundnut-based biscuits and a vitamin-fortified tea drink. Composition of biscuits i.e. 468kcal, 17.4g protein, 25.5g fat, 180mg Calcium, riboflavin 0.23, vitamin A 0micrograms, vitamin C 0mg per 100g. Composition of tea: 78 kcal, 2.9g protein, 1.6g fat, Calcium 95 micrograms, vitamin C	All subjects had free daily access to a sophisticated level of health care provided by a resident midwife and pediatrician at a clinic within the village. Women of childbearing age also attended ante- and postnatal clinics at 6-wk intervals. Antenatal care included: clinical examination and interview; monitoring of blood pressure, fetal growth (fundal height), fetal heart, and	Low birth weight, small-for-gestational age and birth weight.

		10mg per 100g. Maximum intake of supplement biscuit was limited to 3x65g biscuits and 380g tea in the dry season (1209kcal total) and in the hungry season it was raised to 4x65g biscuits and 380g tea (1513kcal total). Food was consumed under direct supervision. In Ramadan, the supplement was provided at night. Women were enrolled as soon as pregnancy and mean duration of supplementation was 24 weeks.	fetal presentation; immunization against tetanus; screening of urine samples for protein and urinary tract infections; and screening of blood samples for anemia and malaria. All women were prescribed daily iron (47 mg Fe as ferrous sulfate) and folate and immediate treatment was offered for all diagnosed infections. Women in whom major obstetrical difficulties were anticipated were referred to hospital for delivery.	
Ross 1985[10]	South Africa	Group 3: High bulk supplements: mixture of beans and maize in a 1.2:1 ratio as mush with added vitamins. This closely resembled the diet normally eaten by most Kwa-Mashu women except that the beans increased the protein content to a higher than usual level. Protein: 36g vegetable, 3247kJ (776kcal) energy, 40mg ascorbic acid.<br><br>Group 4: Low Bulk supplement, a porridge containing 100 g dry skimmed milk, maize flour, vitamins and minerals. It differed from the group 3 supplement in its 36g of animal protein and in its	Group I: No supplementation provided Group II: Zinc supplementation provided (30-90mg zinc gluconate daily) All of the women in the study had routine medical care in the Kwa-Mashu antenatal clinic.	Birth weight only.

	Tontisirin 1986[11]	Thailand	higher levels of several vitamins and calcium. Protein: 36g animal, 8g vegetable. 2927kJ (700kcal) Energy. Supplementation was provided from 20 weeks of pregnancy to birth on weekdays. Group I received a mix of soybean, mungbean, sesame and sugar coming to, on average, 384 kcal energy, 9.1g fat and 15g protein. Group II received a mix of rice, dried shrimp, groundnut, sugar and oil coming to, on average, 348 kcal energy, 15.6g fat and 13.1g protein. Supplementation was only to be consumed in addition to the usual three meals a day. Supplements were delivered at the regional MCH Center.	Provided with no supplements. No other details granted.	Birth weight, birth length and head circumference.
**Food Distribution Program**	Ashorn 2015 [12,13]	Malawi	Participants received intermittent preventive malaria treatment and tailor-made SQ-LNS. Daily dose of 20g to provide the same micronutrients as MMN, 4 additional minerals (calcium, phosphorus, potassium, magnesium), protein and fat providing 118kcal of energy. Fed during pregnancy and until 6 months postpartum.	Received standard of care (Malawian antenatal care) including supplementation of calcium 200mg, iron 60mg, folic acid 400µg and 2 doses of malaria treatment of sulfadoxine-pyrimethamine (3 tablets of 500mg sulfadoxine and 25mg pyrimethamine PO at enrollment and then again between 28 and 34 weeks. Children and mothers in the IFA group received no supplementation.	Stillbirth, perinatal mortality, neonatal mortality, infant mortality, maternal mortality, anemia, low birth weight, preterm weight, small-for-gestational age, birth weight, birth length, head circumference,

				stunting and underweight.
Frith 2015[14]	Bangladesh	The supplement contained 2.5 MJ/day (598kcal), 6 days a week composed of rice, lentils, molasses and oil, which was provided from 9 weeks of pregnancy to 6 months postpartum.	The supplement contained rice, lentils, molasses and oil, contained 2.5 MJ, 6 days a week (29% of recommended energy intake), 25% of which was vegetable protein. Supplement was provided from 20 weeks of pregnancy to 6 months postpartum.	Low birth weight, birth weight, birth length, head circumference,
Johnson 2016[15]	Gambia	1 arm: Protein Energy (PE were provided with 746 kcal/day of energy from protein and lipids)	FeFol (iron and folate supplementation) as usual prenatal care was given	Low birth weight, preterm birth and small-for-gestational-age.
Leroy 2016[16]	Burundi	The food component aimed to increase household food security with multinutrient food rations and corn-soy blend (CSB) and fortified vegetable oil in household and individual rations. T24: program benefits during pregnancy and until 23.9 months of the child. T18: program benefits received during pregnancy and until 18 months of child's age.	The control group did not receive any program benefits but continued to have access to the standard care provided by the Ministry of Health. Other details of the control were not specified.	Anemia, stunting, hemoglobin levels and iron deficiency anemia of child.
Mridha 2016[17]	Bangladesh	Participants received intermittent preventive malaria treatment and tailor-made SQ-LNS. Daily dose of 20g to provide the same micronutrients as MMN, 4 additional minerals (calcium,	Three arms that were grouped into one: 1) control group in which women were given IFA once daily during pregnancy and once every other day for 3 months postpartum period. 2) Child-only LNS group: women	Miscarriage, stillbirths, perinatal mortality, maternal mortality, low birth weight, preterm birth, small-for-

			phosphorus, potassium, magnesium), protein and fat providing 118kcal of energy. Fed during pregnancy and until 6 months postpartum.	received 1 tablet of 60 mg Fe and 400 mg folic acid/day during pregnancy and every alternate day during the first 3 months postpartum and their children received LNS-C from the ages of 6 to 24 months.<br>3) Child-only micronutrient group: women received IFA daily during pregnancy and every alternate day during the first 3 months postpartum and their children received micronutrient powder from the ages of 6 to 24 months.	gestational age, birth weight, birth length, head circumference, stunting and underweight.
**Interventions for Obesity Prevention**	Liu 2016[18]	China	The education intervention based on the TTM theory was provided after the basic education given to all women. Intervention women received three face-to-face interventions and three follow-up phone calls which were developed based on the Transtheoretical Mode (TTM).<br>TTM is an integrative and biopsychosocial model to conceptualize an intentional behavior change's process that unfolds overtime through a series of stages. It consists of 5 stages in successful behavior change: pre-contemplation, contemplation, preparation, action, and maintenance.	At the first prenatal check, the investigator provided routine health education about the effects of excessive gestational weight on pregnancy outcomes and explained the pattern of ideal weekly gain and overall maternal weight gain based on participants' calculated BMI. The maternal health handbook was distributed as a medical record at the first prenatal check which recorded the weight of each prenatal visit, which was a routine prenatal care. Participants were weighed at the postpartum visit which occurred 42 days after delivery.	Macrosomia and birth weight.

Aşcı 2016[19]	Turkey	Four meetings regarding healthy lifestyle, nutrition, exercise, and weight follow-up supplemented with health training and brochures. Maternal anthropometry was performed. The women were provided with praise and those that were not able to meet objectives had their short comings reviewed and provided intensive consultancy (reemphasis of recommendations and added phone consultancy) was provided. In weeks 12 to 15, the focus was on healthy life and health practices. In weeks 16 to 18, the focus was on physical activity ad exercises. In weeks 20-24, interviews regarding nutrition were held i.e. meal frequency, size, and content. On week 37, only weights were followed up and target achievement was reviewed and the intervention was ended. At 6 weeks postpartum obstetric and neonatal outcomes were measured.	Consultancies mostly consist of subjects such as pregnancy complaints, scope of antenatal care, tests to be performed, birth, post-partum period, and circumstances that might pose danger during pregnancy, There is no standard training and consultancy. Women in the standard care group (control) are followed up at least four times by midwives or nurses and weights are measured. Consultancy is granted for pregnancy complaints, tests, birth and post-partum period only	Birth weight and birth length.

Table S4. Summary of Meta-Analysis

Outcome	Strategy	Overall effect, sample size, heterogeneity	Analysis number of studies	Subgroup effects estimate, sample size, heterogeneity
		**Balanced Energy Protein**		
Primary outcomes				
Stillbirth	Balanced Energy Protein vs. control	RR 0.39; 95% CI 0.19 to 0.80, n=1913, Chi² = 0.45, (P = 0.80); I² = 0%	BEP studies (n=3)[1-3]	
	BEP in Africa vs. BEP in Asia	Overall: RR 0.39; 95% CI 0.19 to 0.80, n=1913, Chi² = 0.45, (P = 0.80); I² = 0%	Africa (n=1)[1]<br>Asia (n=1) [2]<br>South America (n=1) [3]	Africa: RR 0.45; 95% CI 0.20 to 1.04, n=1446, NA<br>Asia: RR 0.33; 95% CI 0.01 to 7.45, n=24, NA<br>South America: RR: 0.25. 95% CI 0.05, 1.17, n= 443, NA
	BEP in second trimester vs. BEP in third trimester vs. BEP in whole pregnancy	Overall: RR 0.39; 95% CI 0.19 to 0.80, n=1913, Chi² = 0.45, (P = 0.80); I² = 0%	Second trimester (n=1) [1]<br>Third trimester (n=1) [3]<br>Whole pregnancy (n=1) [2]	Second trimester: RR 0.45; 95% CI 0.20 to 1.04, n=1446, NA<br>Third trimester: RR: 0.25. 95% CI 0.05, 1.17, n= 443, NA<br>Whole pregnancy: RR 0.33; 95% CI 0.01 to 7.45, n=24, NA
	BEP in urban vs BEP in rural settings	Overall: RR 0.39; 95% CI 0.19 to 0.80, n=1913, Chi² = 0.45, (P = 0.80); I² = 0%	Urban (n=1) [2]<br>Rural (n=2) [1] [3]	Urban: RR 0.33; 95% CI 0.01 to 7.45, n=24, NA<br>Rural: RR 0.40; 95% CI 0.19 to 0.82, n=1889, NA
Secondary outcomes				
Birth weight	Balanced Energy protein vs. control	MD 107.28; 95% CI 68.51 to 146.04, n=2190, Chi² = 13.84, (P =0.13); I² = 35%	BEP studies (n=8) [1-4,9-11]	
Birth length	Balanced Energy protein vs. control	MD 0.28; 95% CI -0.36 to 0.92, n=67, Chi² = 0.54, (P = 0.77); I² = 0%	BEP studies (n=2) [2,11]	
Low birth weight	Balanced Energy protein vs. control	RR 0.60; 95% CI 0.41 to 0.86, n=1830, Chi² = 2.74, (P = 0.25); I² = 27%	BEP studies (n=3) [1,2,9]	

Preterm Birth	Balanced Energy protein vs. control	RR 0.86; 95% CI 0.50 to 1.46, n=467, Chi² = 0.37, (P = 0.54); I² = 0%	BEP studies (n=2) [2,3]	
Small-for-gestational age	Balanced Energy protein vs. control	RR 0.71; 95% CI 0.54 to 0.94, n=1844, Chi² = 6.32, (P = 0.28); I² = 21%	BEP studies (n=5) [1-3,9]	
**Food Distribution Programs**				
Primary outcomes				
Perinatal mortality	Food distribution program vs. control	RR 0.67; 95% CI 0.41 to 1.09, n=4852, Chi² = 1.13, (P = 0.29); I² = 11%	FDP studies (n=2)[12,17]	
	FDP in Africa vs. FDP in Asia vs FDP in South America	Overall: RR 0.67; 95% CI 0.41 to 1.09, n=4852, Chi² = 1.13, (P = 0.29); I² = 11%	Africa (n=1) [12] Asia (n=1) [17]	Africa: RR 0.45; 95% CI 0.19 to 1.09, n=841, NA Asia: RR 0.78; 95% CI 0.47 to 1.32, 4011, NA
Secondary outcomes				
Low birth weight	Food distribution program vs. control	RR 0.92; 95% CI 0.84 to 1.00, n=5552, Chi² = 0.78, (P = 0.85); I² = 0%	FDP studies (n=4) [12,14,15,17]	
Maternal mortality	Food distribution program vs. control	RR 0.41, 95% CI 0.07 to 2.49, n=4925, Chi² = 0.07, (P = 0.79); I² = 0%	FDP studies (n=2) [13,17]	
Preterm birth	Food distribution program vs. control	RR 0.92; 95% CI 0.78 to 1.10, n=4608, Chi² = 1.16, (P = 0.56); I² = 0%	FDP studies (n=3) [12,15,17]	
Small-for-gestational age	Food distribution program vs. control	RR 0.94; 95% CI 0.89 to 1.00, n=4511, Chi² = 0.36, (P = 0.84); I² = 0%	FDP studies (n=3) [12,15,17]	
Birth weight	Food distribution program vs. control	MD 46.00; 95% CI 45.10 to 46.90, n=5272, Chi² = 0.37, (P = 0.83); I² = 0%	FDP studies (n=3) [13,14,17]	
Birth length	Food distribution program vs. control	MD 0.20, 95% CI 0.20 to 0.20, n=5272, Chi² = 1.54, (P = 0.46); I² = 0%	FDP studies (n=3) [13,14,17]	
Head circumference	Food distribution program vs. control	MD 0.07; 95% CI -0.22 to 0.36, n=4490, Chi² = 8.58, (P = 0.003); I² = 88%	FDP studies (n=2) [14,17]	
Stunting	Food distribution program vs. control	RR 0.82; 95% CI 0.71 to 0.94, n=4166, Chi² = 0.13, (P 0.72); I² = 0%	FDP studies (n=2) [12,17]	
Wasting	Food distribution program vs. control	RR 0.87; 95% CI 0.78 to 0.97, n=3449, Chi² = 0.00, (P = 0.95); I² = 0%	FDP studies (n=2) [12,17]	

Underweight	Food distribution program vs. control	RR 0.84; 95% CI 0.63 to 1.13, n=4174, Chi^2 = 1.70, (P = 0.19); I^2 = 41%	FDP studies (n=2) [12,17]
**Obesity Prevention**			
Primary outcomes			
Birth weight	Obesity prevention program vs. control	MD -195.57, 95% CI -349.46 to -41.68, n=180, Chi^2 = 0.04, (P = 0.84); I^2 = 0%	Obesity prevention studies (n=2) [18,19]

Table S5. Summary of Findings (SoF) Table of Balanced Energy Protein (BEP)

Balanced Energy Protein compared to Control (Newly Added) for Maternal, Neonatal and Childhood Outcomes					
**Patient or population: Mothers, Neonates and Children**<br>**Setting: Low- and Middle- Income Countries**<br>**Intervention: Balanced Energy Protein**<br>**Comparison: Control**					
	**Anticipated absolute effects*** **(95% CI)**				
	**Risk with Control (Newly Added)**	**Risk with Balanced Energy Protein**			
Stillbirth	28 per 1,000	**11 per 1,000** (5 to 22)	**RR 0.39** (0.19 to 0.80)	1913 (3 RCTs)	⊕⊕◯◯ LOW [a,b]
Perinatal Mortality	59 per 1,000	**30 per 1,000** (18 to 50)	**RR 0.50** (0.30 to 0.84)	1446 (1 RCT)	⊕◯◯◯ VERY LOW [a,b,c]
Neonatal Mortality	39 per 1,000	**23 per 1,000** (13 to 42)	**RR 0.58** (0.32 to 1.06)	1446 (1 RCT)	⊕◯◯◯ VERY LOW [a,b,d]
Infant Mortality	38 per 1,000	**38 per 1,000** (22 to 64)	**RR 1.00** (0.59 to 1.68)	1446 (1 RCT)	⊕⊕⊕◯ MODERATE [b]
Low Birth weight	171 per 1,000	**103 per 1,000** (70 to 147)	**RR 0.60** (0.41 to 0.86)	1830 (3 RCTs)	⊕⊕◯◯ LOW [e,f]
Preterm Birth	111 per 1,000	**96 per 1,000** (56 to 162)	**RR 0.86** (0.50 to 1.46)	467 (2 RCTs)	⊕◯◯◯ VERY LOW [g,h]

***The risk in the intervention group** (and its 95% confidence interval) is based on the assumed risk in the comparison group and the **relative effect** of the intervention (and its 95% CI).

CI: Confidence interval; **RR:** Risk ratio

GRADE Working Group grades of evidence
High certainty: We are very confident that the true effect lies close to that of the estimate of the effect
Moderate certainty: We are moderately confident in the effect estimate: The true effect is likely to be close to the estimate of the effect, but there is a possibility that it is substantially different
Low certainty: Our confidence in the effect estimate is limited: The true effect may be substantially different from the estimate of the effect
Very low certainty: We have very little confidence in the effect estimate: The true effect is likely to be substantially different from the estimate of effect

Table S6. Summary of Findings of Food Distribution Program (FDP)

Food Distribution Program compared to Control for Maternal, Neonatal and Childhood Outcomes

Patient or population: Mothers, Neonates and Children
Setting: Low- and Middle- Income Countries
Intervention: Food Distribution Program
Comparison: Control

	**Anticipated absolute effects*** **(95% CI)**				
	**Risk with Control**	**Risk with Food Distribution Program**			
Perinatal Mortality	24 per 1,000	**16 per 1,000** (10 to 26)	**RR 0.67** (0.41 to 1.09)	4852 (2 RCTs)	⊕⊕◯◯ LOW [a,b]
Neonatal Mortality	42 per 1,000	**19 per 1,000** (8 to 44)	**RR 0.46** (0.20 to 1.04)	841 (1 RCT)	⊕⊕◯◯ LOW [c,d]
Infant Mortality	2 per 1,000	**1 per 1,000** (0 to 20)	**RR 0.34** (0.01 to 8.41)	841 (1 RCT)	⊕⊕⊕◯ MODERATE [d]
Low Birth Weight	340 per 1,000	**313 per 1,000** (286 to 340)	**RR 0.92** (0.84 to 1.00)	5552 (4 RCTs)	⊕⊕⊕◯ MODERATE [e]
Preterm birth	128 per 1,000	**118 per 1,000** (100 to 141)	**RR 0.92** (0.78 to 1.10)	4608 (3 RCTs)	⊕⊕⊕◯ MODERATE [f]

***The risk in the intervention group** (and its 95% confidence interval) is based on the assumed risk in the comparison group and the **relative effect** of the intervention (and its 95% CI).

CI: Confidence interval; **RR:** Risk ratio

GRADE Working Group grades of evidence
High certainty: We are very confident that the true effect lies close to that of the estimate of the effect
Moderate certainty: We are moderately confident in the effect estimate: The true effect is likely to be close to the estimate of the effect, but there is a possibility that it is substantially different
Low certainty: Our confidence in the effect estimate is limited: The true effect may be substantially different from the estimate of the effect
Very low certainty: We have very little confidence in the effect estimate: The true effect is likely to be substantially different from the estimate of effect

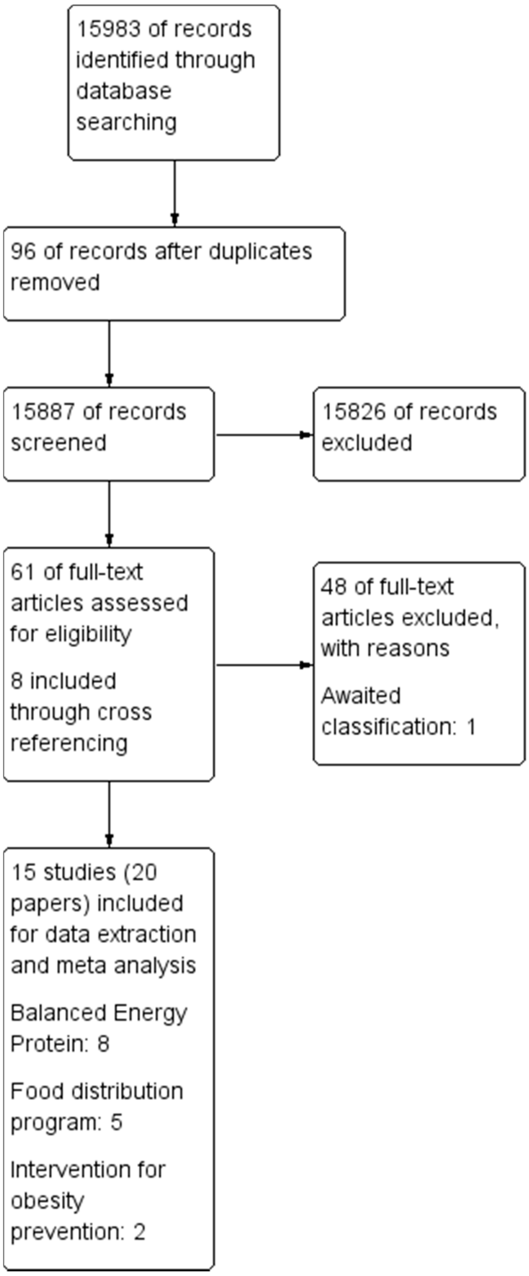

Figure S1. Search Flow Diagram.

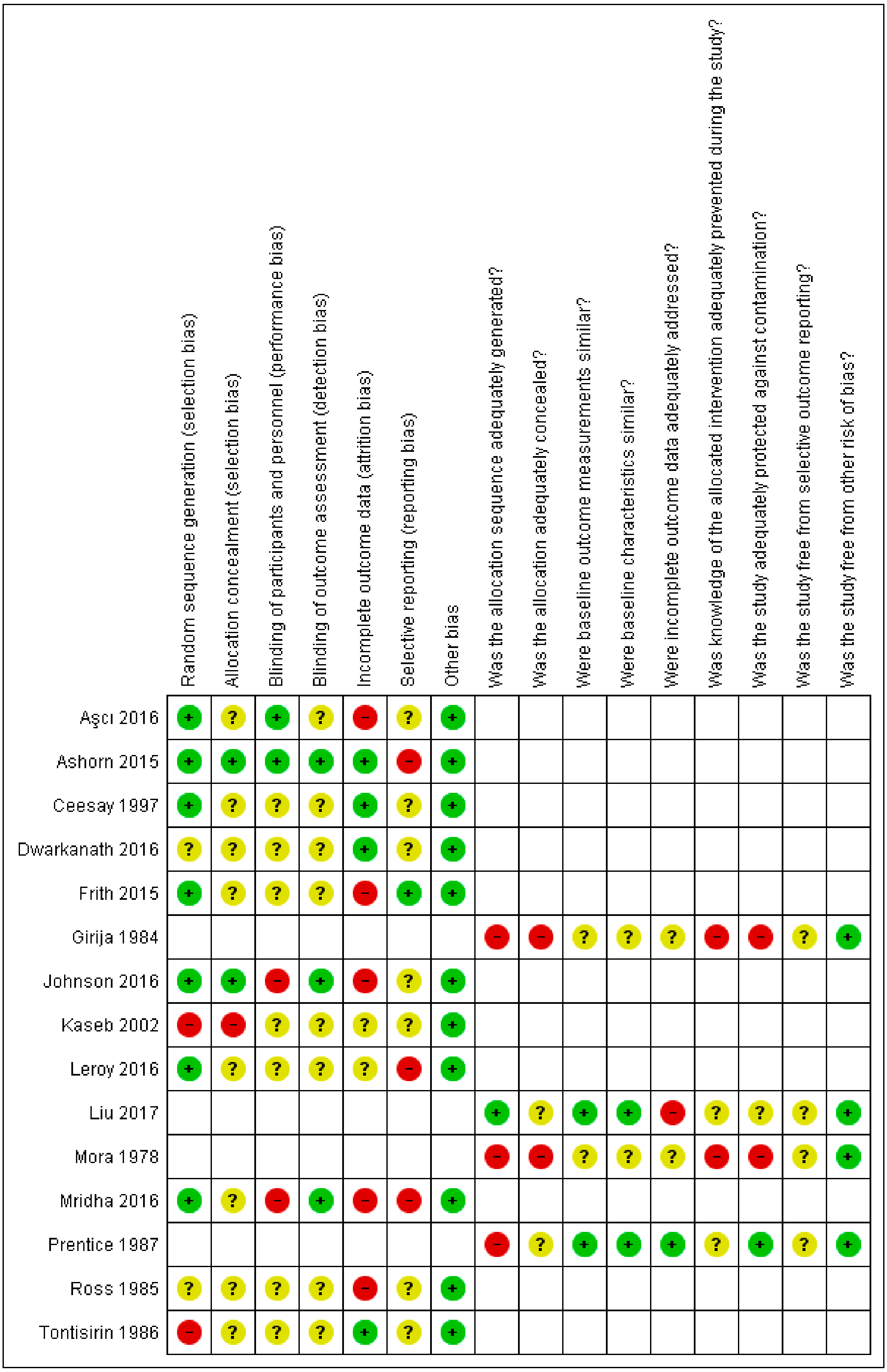

Figure S2. Risk of Bias Summary

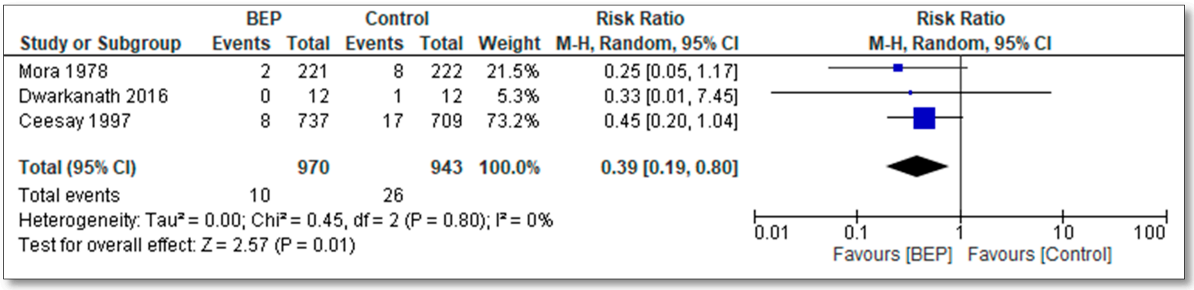

Figure S3. Forest plot of comparison: Balanced Energy Protein vs. Control, outcome: Stillbirth.

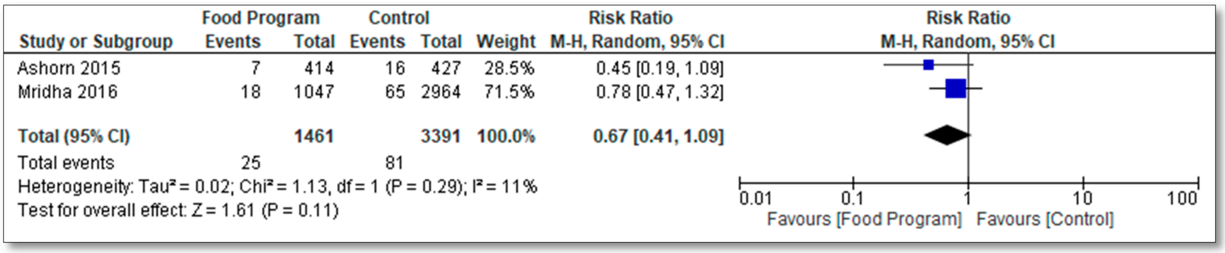

Figure S4. Forest plot of comparison: Food Program vs. Control, outcome: Perinatal Mortality.

References

1. Ceesay, S.M.; Prentice, A.M.; Cole, T.J.; Foord, F.; Weaver, L.T.; Poskitt, E.M.; Whitehead, R.G. Effects on birth weight and perinatal mortality of maternal dietary supplements in rural Gambia: 5 year randomised controlled trial. *BMJ (clinical research ed.)* **1997**, *315*, 786-790.
2. Dwarkanath, P.; Hsu, J.W.; Tang, G.J.; Anand, P.; Thomas, T.; Thomas, A.; Sheela, C.N.; Kurpad, A.V.; Jahoor, F. Energy and Protein Supplementation Does Not Affect Protein and Amino Acid Kinetics or Pregnancy Outcomes in Underweight Indian Women. *Journal of nutrition* **2016**, *146*, 218-226, doi:10.3945/jn.115.218776.
3. Girija, A.; Geervani, P.; Rao, G.N. Influence of dietary supplementation during pregnancy on lactation performance. *J Trop Pediatr* **1984**, *30*, 79-83, doi:10.1093/tropej/30.2.79.
4. Kaseb, F.; Kimiagar, M.; Ghafarpoor, M.; Valaii, N. Effect of traditional food supplementation during pregnancy on maternal weight gain and birthweight. *Int J Vitam Nutr Res* **2002**, *72*, 389-393, doi:10.1024/0300-9831.72.6.389.
5. Mora, J.O.; de Paredes, B.; Wagner, M.; de Navarro, L.; Suescun, J.; Christiansen, N.; Herrera, M.G. Nutritional supplementation and the outcome of pregnancy. I. Birth weight. *American journal of clinical nutrition* **1979**, *32*, 455-462, doi:10.1093/ajcn/32.2.455.
6. O. Mora, J.; Clement, J.; Christiansen, N.; Suescun, J.; Wagner, M.; G. Herrera, M. Nutritional Supplementation and the Outcome of Pregnancy. III. Perinatal and Neonatal Mortality. *Obstetrical & Gynecological Survey* **1978**, *34*, 437-439, doi:10.1097/00006254-197906000-00005.
7. Vuori, L.; Christiansen, N.; Clement, J.; Mora, J.O.; Wagner, M.; Herrera, M.G. Nutritional supplementation and the outcome of pregnancy. II. Visual habituation at 15 days. *American journal of clinical nutrition* **1979**, *32*, 463-469, doi:10.1093/ajcn/32.2.463.
8. Vuori, L.; de Navarro, L.; Christiansen, N.; Mora, J.O.; Herrera, M.G. Food supplementation of pregnant women at risk of malnutrition and their newborns' responsiveness to stimulation. *Developmental medicine and child neurology* **1980**, *22*, 61-71.
9. Prentice, A.M.; Cole, T.J.; Foord, F.A.; Lamb, W.H.; Whitehead, R.G. Increased birthweight after prenatal dietary supplementation of rural African women. *The American journal of clinical nutrition* **1987**, *46*, 912-925, doi:10.1093/ajcn/46.6.912.
10. Ross, S.M.; Nel, E.; Naeye, R.L. Differing effects of low and high bulk maternal dietary supplements during pregnancy. *Early Hum Dev* **1985**, *10*, 295-302.

11. Tontisirin, K.; Booranasubkajorn, U.; Hongsumarn, A.; Thewtong, D. Formulation and evaluation of supplementary foods for Thai pregnant women. *The American journal of clinical nutrition* **1986**, *43*, 931-939, doi:10.1093/ajcn/43.6.931.
12. Ashorn, P.; Alho, L.; Ashorn, U.; Cheung, Y.B.; Dewey, K.G.; Harjunmaa, U.; Lartey, A.; Nkhoma, M.; Phiri, N.; Phuka, J., et al. The impact of lipid-based nutrient supplement provision to pregnant women on newborn size in rural Malawi: a randomized controlled trial. *American journal of clinical nutrition* **2015**, *101*, 387-397, doi:10.3945/ajcn.114.088617.
13. Ashorn, P.; Alho, L.; Ashorn, U.; Cheung, Y.B.; Dewey, K.G.; Gondwe, A.; Harjunmaa, U.; Lartey, A.; Phiri, N.; Phiri, T.E., et al. Supplementation of Maternal Diets during Pregnancy and for 6 Months Postpartum and Infant Diets Thereafter with Small-Quantity Lipid-Based Nutrient Supplements Does Not Promote Child Growth by 18 Months of Age in Rural Malawi: a Randomized Controlled Trial. *Journal of nutrition* **2015 (b)**, *145*, 1345-1353, doi:10.3945/jn.114.207225.
14. Frith, A.L.; Naved, R.T.; Persson, L.A.; Frongillo, E.A. Early prenatal food supplementation ameliorates the negative association of maternal stress with birth size in a randomised trial. *Maternal & child nutrition* **2015**, *11*, 537-549, doi:10.1111/mcn.12047.
15. Johnson, W.; Darboe, M.K.; Sosseh, F.; Nshe, P.; Prentice, A.M.; Moore, S.E. Association of prenatal lipid-based nutritional supplementation with fetal growth in rural Gambia. *Maternal & child nutrition* **2017**, *13*, doi:10.1111/mcn.12367.
16. Leroy, J.L.; Olney, D. Tubaramure, a Food-Assisted Integrated Health and Nutrition Program in Burundi, Increases Maternal and Child Hemoglobin Concentrations and Reduces Anemia: A Theory-Based Cluster-Randomized Controlled Intervention Trial. **2016**, *146*, 1601-1608, doi:10.3945/jn.115.227462.
17. Mridha, M.K.; Matias, S.L.; Chaparro, C.M.; Paul, R.R.; Hussain, S.; Vosti, S.A.; Harding, K.L.; Cummins, J.R.; Day, L.T.; Saha, S.L., et al. Lipid-based nutrient supplements for pregnant women reduce newborn stunting in a cluster-randomized controlled effectiveness trial in Bangladesh. *The American journal of clinical nutrition* **2016**, *103*, 236-249, doi:10.3945/ajcn.115.111336.
18. Liu, Y.Q.; Liu, Y.; Hua, Y.; Chen, X.L. Effect of diet and exercise intervention in Chinese pregnant women on gestational weight gain and perinatal outcomes: A quasi-experimental study. *Applied nursing research : ANR* **2017**, *36*, 50-56, doi:10.1016/j.apnr.2017.05.001.
19. Aşcı, Ö.; Rathfisch, G. Effect of lifestyle interventions of pregnant women on their dietary habits, lifestyle behaviors, and weight gain: a randomized controlled trial. *Journal of health, population, and nutrition* **2016**, *35*, 7, doi:10.1186/s41043-016-0044-2.
